# Use of serum-free media for peripheral blood mononuclear cell culture and the impact on T and B cell readouts

**DOI:** 10.3389/ftox.2024.1462688

**Published:** 2024-11-05

**Authors:** Stella Cochrane, Ouarda Saib, David Sheffield

**Affiliations:** Safety and Environmental Assurance Centre (SEAC), Unilever, Colworth Science Park, Sharnbrook, United Kingdom

**Keywords:** PBMC, immune modulation, *in vitro*, animal-free, serum-free, T cell, B cell

## Abstract

**Introduction:**

As part of a wider programme of work developing next-generation risk assessment approaches (NGRA) using non-animal methods (NAMs) for safety assessment of materials, Unilever SEAC is exploring the use of a peripheral blood mononuclear cell (PBMC) system to investigate how cells from different arms of the human immune system are impacted by different treatments. To maximise human relevance, the cell cultures are supported by human serum, but this came with some challenges, including an inability to measure induced levels of immunoglobulins due to high background levels. Therefore, a study comparing use of human sera containing media with three different chemically defined serum-free media was undertaken.

**Materials and Methods:**

PBMC were isolated from healthy donors and cultured in the absence (media alone) or presence of stimulation reagents (CpG-ODN plus IL-15, Pokeweed Mitogen (PWM) or Cytostim (CS)), in RPMI plus human serum, AIM-V, CTS OpTmizer T cell expansion SFM or X-VIVO 15 media. T cell (CD4^+^ and CD8^+^) and B cell proliferation and viability were measured after 6 days, along with levels of total IgG in the cell culture supernatants.

**Results:**

Each of the serum-free media tested supported good levels of viable and proliferating T cells and B cells over the 6 days of culture, with only a few, small differences across the media, when there was no stimulation. They also enabled detection of a stimulation-evoked increase in IgG levels. There were however some differences in the viability and proliferation responses of T and B cells, to different stimuli, across the different media.

**Discussion:**

The serum-free media formulations tested in this study offer defined systems for. measuring B cell IgG responses, *in vitro*, in either a ‘T cell-independent’ (CpG + IL-15) or “T cell-dependent” (PWM or CS) manner and for assessing B cell proliferation, particularly in response to a “T cell-independent” stimulus. However, there are some characteristics and features endowed by human serum that appear to be missing. Therefore, further work is required to optimise animal-free, chemically defined culture conditions for PBMC based assays for inclusion in tiered safety assessments.

## 1 Introduction

In toxicology, steps are being taken towards more mechanism-focused and relevant human approaches to risk assessment, driving the development of new methods including *in vitro* assays to assess the effects of materials on the human immune system.

As part of a wider programme of work developing next-generation risk assessment approaches (NGRA) using non-animal methods (NAMs) for safety assessment of materials (Carmichael et al., 2022), Unilever SEAC is exploring the use of a peripheral blood mononuclear cell (PBMC) system to investigate how cells from different arms of the human immune system are impacted by different treatments. The PBMC based system in question is described in detail in Cochrane et al. (Cochrane et al., 2024) and, as detailed in that paper, the cell cultures were supported by human serum to address the multiple disadvantages associated with use of foetal bovine serum (van der Valk et al., 2018; van der Valk, 2022) and further enhance human translatability. Details regarding the stimuli used in this paper, including the rationale for their selection and known mechanisms of action are detailed in Cochrane et al., 2024. In brief, CytoStim (CS) activates T cells by cross-linking the TCR with MHCII, while pokeweed mitogen (PWM) was chosen to cover T-cell-dependent activation of B cells. CpG ODN (CpG) with support from IL-15 was chosen for T-cell-independent mechanisms. Cochrane et al., 2024 also provides more information on aspects such as choice of use of PBMCs and selection of cell populations for *ab initio* assessment of exogenous materials. It also discusses the advantages and limitations of the system that are beyond the scope of this paper. This paper focuses on presenting the results and findings of a specific study to evaluate the impact of different culture media on selected cells and read outs. As part of the drive to maximise the reliability and human relevance of such *in vitro* methods, the impact of inclusion of animal-derived materials in culture systems has come under increasing scrutiny and is of particular concern in studies of human immune responses due to the immunogenic potential of exogenic materials (Witzeneder et al., 2013). The use of human serum did however come with some challenges. Most notable was the presence of pre-existing human solutes that could potentially confound subsequent measurement in culture media. One issue encountered was an inability to measure induced levels of immunoglobulins with the 2 B cell stimuli used due to high background levels in human serum. In response to this, a study comparing use of human sera containing media with three different chemically defined serum-free media was undertaken. Use of chemically defined media is becoming increasingly important, as not only does this address the aforementioned issues associated with use of animal-derived materials, but also factors such as a lack of defined, standardised composition of human serum. This paper presents the results and findings of this work.

## 2 Materials and methods

### 2.1 Experimental method

All experimental work was carried out by the CRO Celentyx Limited. Peripheral blood mononuclear cells (PBMC) were isolated from eight healthy donors through Ficoll-Paque PLUS (GE Healthcare; 11778538) density centrifugation. All samples were obtained with informed consent and approval from the London–South East Research Ethics Committee (REC reference: 16/LO/0601).

PBMC were isolated and used fresh, cultured at 2 × 10^5^ cells per well in the absence (media alone) or presence of the following stimulation reagents at the indicated concentrations:

• CpG-ODN at 0.2 μM (Invivogen; tlrl-2006) plus IL-15 at 15 ng per mL (Immunotools 11340157) (CpG + IL15)
• Pokeweed Mitogen (PWM) at 5.0 μg per mL (Sigma-Aldrich L8777)
• Cytostim (CS) at 1.2 μL per mL (Miltenyi Biotec 130–092-173)

Except for PWM, which was reconstituted in PBS, the stimulation reagents were reconstituted in HyClone HyPure WFI Quality Water (GE LifeSciences). The final use concentrations indicated above were selected based on available information regarding their impact on PBMCs and further information can be found in Cochrane et al., 2024. CS solutions were stored at 4°C and the others at −20°C.

Cultures were set up in 96-well round bottom plates (Sarstedt) for 6 days at 37°C, 5% CO_2_ in the following different media:

• RPMI 1640 Medium (with sodium bicarbonate and L-glutamine, Sigma-Aldrich) with 1% (v/v) penicillin/streptomycin (Sigma-Aldrich) and 5% (v/v) heat-inactivated human serum (Tissue Solutions Ltd.) Referred to as RPMI + HS in this paper.
• AIM-V media (ThermoFisher 12055) with 1% penicillin/streptomycin. Referred to as AIM-V in this paper.
• CTS OpTmizer T cell expansion SFM (Fisher Scientific 10781764) with 1% penicillin/streptomycin and 1% GlutaMAX™ supplement (Thermo Fisher; 35050061). Referred to as CTS in this paper.
• X-VIVO 15 media (Lonza 04-418Q) with 1% penicillin/streptomycin. Referred to as X-VIVO in this paper.

For proliferation measurements, PBMCs were labelled with cell proliferation dye eFluor 450^®^ (Thermo Fisher. Cat. No. 65–0842-85). After 6 days of cell culture, the plates were centrifuged to pellet cells and supernatants removed and stored at −20°C for subsequent analysis of IgG levels.

The cells were then stained with anti-human CD3 APC, CD4 PE/Cy7, CD8 BV510 and CD19 PE (all BioLegend, 317318, 317414, 344732, 302208, respectively), as well as Zombie green viability dye (BioLegend, 423112), and then fixed using the FoxP3 staining buffer set (BD Biosciences, 560098).

Following fixation, which was done by diluting the fix concentrate 10-fold in distilled water and adding 100 μL of fix buffer per well, incubation for 10 min at room temperature was performed in the dark. The cells were then washed and resuspended in PBS. AccuCheck Counting Beads (ThermoFisher, PCB100) were added to each sample immediately before running on a BD.

FACScan cytometer. Results were analysed using FlowJo 10.3. The gating for T cells and B cells was as follows: FSc vs. SSc (Cells) > PulseWidth vs. FSc (singlets) > viability vs. SSc (viable cells) > CD3 for T cells plus CD4 or CD8 and CD19 for B cells vs. SSc.

Proliferation was calculated by examining the ‘divided’ peaks and where proliferated counts are reported, this is the number of cells in the ‘divided’ gate, divided by the cell count (viability dye was used to exclude dead cells) multiplied by 100 to give a percentage. Counting beads were used to enumerate absolute numbers per well.

Levels of total IgG in the cell culture supernatants were measured by ELISA (Thermo Fisher 88–50550–88) following manufacturer’s instructions. Samples cultured in AIM-V, CTS or X-VIVO media were diluted one in five for analysis, whilst samples cultured in RPMI + HS were diluted one in 5,000 for analysis. Samples were measured using an iMARK™ microplate reader and MPM software.

For each donor, isolated PBMCs were plated out in triplicate for each treatment condition (e.g., unstimulated, or stimulation with CS) in each media, providing three replicate measurements for each parameter, for each treatment condition, in each media per donor.

### 2.2 Culture media review

A desk-based review was undertaken to understand, as fully as possible, the components of the three serum-free media selected for this study: AIM-V media (ThermoFisher; 12055), CTS OpTmizer T cell expansion SFM (Fisher Scientific; 10781764) and X-VIVO 15 media (Lonza; 04-418Q). Detailed investigations enabled us to confirm the “animal-free” status of these media through conversations with the suppliers to supplement information found on the manufacturer website, and further understand any differences in components across the media that could impact upon the results obtained.

The key components of the different media used, and manufacturers details are captured in Table 1.

**TABLE 1:** Key component and manufacturers information for the four media used in this study.

Medium	Manufacturer	Indicated cell type/Application	Classification	Additives	Included antibiotics	Inorganic salts	Other materials present
RPMI 1640 (with sodium bicarbonate and L-glutamine)	Sigma-Aldrich	Mammalian cells	Animal Origin-free	Sodium Bicarbonate, phenol red, L-glutamine	No antibiotics	-	-
AIM V	ThermoFisher	Monocytes, Dendritic Cells, T Cells, Hybridomas, PBMCs, Fibroblasts, Macrophages, Myeloma Cells, Lymphocytes	Serum-free, Xeno-free	Glutamine, Phenol Red	streptomycin sulphate; gentamicin sulphate	-	supplemented with purified HSA, transferrin, insulin; a proprietary mixture of purified factors (including cholesterol)
CTS OpTmizer T cell expansion SFM	Fisher Scientific	T Cells (CD4^+^, CD8^+^, polyclonal, or antigen-specific T cells)	Serum-free, Xeno-free	HEPES, Phenol Red, Sodium Pyruvate, Sodium Bicarbonate	No antibiotics	Calcium, Magnesium	HSA
X-VIVO 15 media	Lonza	proliferation of isolated CD3^+^ cells, peripheral blood lymphocytes (PBL), and tumour infiltrating lymphocytes (TIL). Cultivation of human monocytes, dendritic cells, macrophages, PBL, granulocytes; natural killer NAK, lymphokine-activated killer (LAK) cells	Chemically defined, serum-free	L-glutamine, and phenol red	gentamicin	-	human albumin, recombinant human insulin; pasteurised human transferrin

### 2.3 Statistics

To compare the results from each stimulation level against the unstimulated results, or results in a serum-free media against RPMI + HS, a linear model was fit to the logged values using the Mixed Procedure in SAS 9.4 (The SAS Institute). Individual donor effects were accounted for using fixed effects by subtracting the relative mean effect for each donor, and the stimulation level means were compared using estimate statements. Statistical tests were at the 5% confidence level.

## 3 Results

The focus of this paper is to convey the impact of different media types on readouts. The data in this section is therefore presented to enable easy comparison of effects across media and to emphasise this aspect of the study. Additionally, we have provided figures in the (Supplementary Figures S1-S4) to enable comparison of the effects of different stimuli delivered in a single media type.

To address potential difficulties in discerning significant changes due to scaling effects, we have incorporated symbols (please refer to figure headings for further details) to indicate these. Additionally, any figures showing numbers of viable or proliferating cells are also provided with log scaling of the Y-axis under to aid in data visualisation (Supplementary Figures S5-S12).

### 3.1 T cell viability and proliferation

**FIGURE 1:**
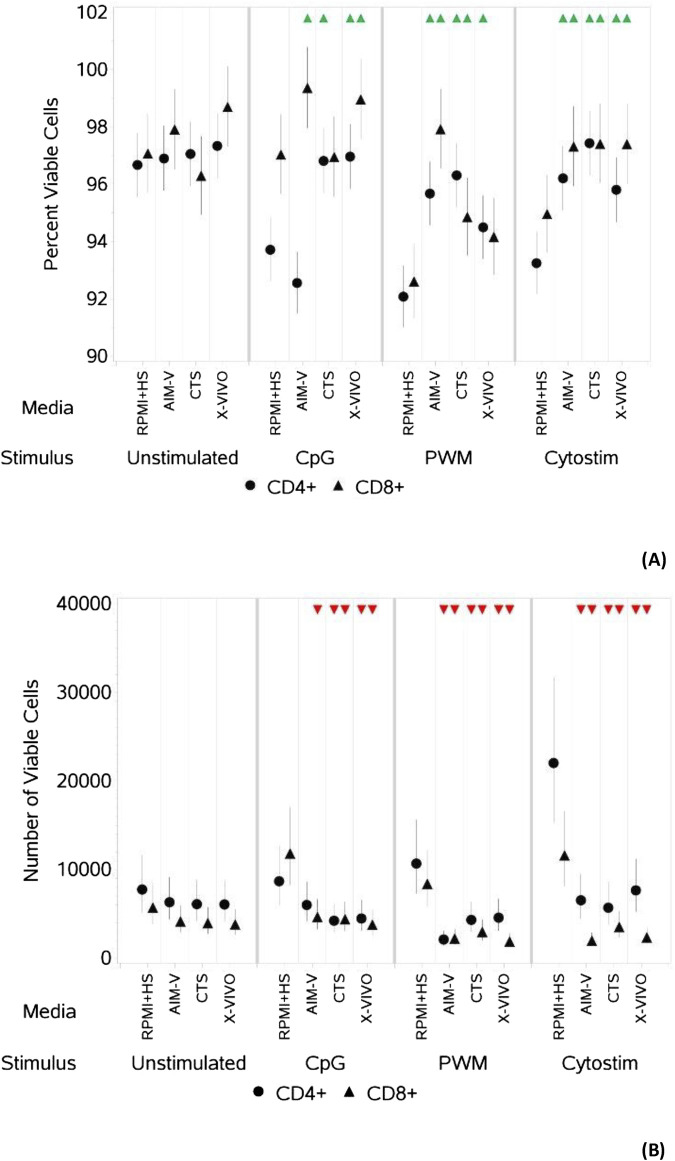
Impact of use of different media on percentage **(A)** and absolute number **(B)** of viable CD4^+^ and CD8^+^ T cells, with and without stimulation. PBMCs were isolated from healthy donors and cultured in the absence (unstimulated) or presence of stimulation reagents (CpG-ODN plus IL-15, Pokeweed Mitogen (PWM) or Cytostim (CS)), in RPMI plus human serum, AIM-V, CTS OpTmizer T cell expansion SFM or X-VIVO 15 media. Viability was measured after 6 days by flow cytometry and Zombie Green viability dye was used along with anti-human CD3, CD4 and CD8 as detailed in the materials and methods section. Red triangles denote a significant decrease compared to RPMI + HS, and green triangles indicate a significant increase. Points and lines represent the mean and 95% confidence interval of the mean after the donor effect has been accounted for (eight donors, each with three measurements), respectively.

The mean percentage of viable CD4^+^ and CD8^+^ T cells essentially remained high (greater than 90%) in all four media both in the absence and presence of stimulants. There was also no statistically significant difference between the percentage and number of viable CD4^+^ and CD8^+^ cells across the different media when unstimulated (Figure 1A).

As summarised in Table 2, there was however a small but significant increase in the percentage of viable CD4^+^ T cells after stimulation with CpG + IL15 in CTS and X-VIVO compared to RPMI + HS, but not in AIM-V. For CD8^+^ T cells an increase was seen in AIM-V and X-VIVO, but not CTS when compared to RPMI + HS. A similar small, but significant increase in the percentage of viable CD4^+^ T cells was seen with PWM and CS in all three serum-free media compared to RPMI + HS. For CD8^+^ T cells the pattern was similar, except with PWM where there was no difference when compared to RPMI + HS in X-VIVO media. Additionally, there was a significantly lower number of viable CD4^+^ T cells after stimulation with CpG + IL15 in CTS and X-VIVO compared to RPMI + HS, but no difference in AIM-V. For CD8^+^ cells a lower number of viable cells was seen in all three serum-free media compared to RPMI + HS with this stimulus. A similar small, but significantly lower number of viable CD4^+^ and CD8^+^ T cells was seen with PWM and CS in all three serum-free media compared to RPMI + HS.

**TABLE 2:** Changes in the percentage and number of viable CD4^+^ and CD8^+^ T cells in different serum-free media compared to RPMI + HS, when unstimulated and stimulated.

Condition	Changes in the percentage and number of viable CD4^+^ and CD8^+^ cells											
	Percent viable cells						Number of viable cells					
	AIM-V CD4^+^ CD8^+^		CTS CD4^+^ CD8^+^		X-VIVO CD4^+^ CD8^+^		AIM-V CD4^+^ CD8^+^		CTS CD4^+^ CD8^+^		X-VIVO CD4^+^ CD8^+^	
Unstimulated	-	-	-	-	-	-	-	-	-	-	-	-
CpG	-	↑	↑	-	↑	↑	-	↓	↓	↓	↓	↓
PWM	↑	↑	↑	↑	↑	-	↓	↓	↓	↓	↓	↓
CS	↑	↑	↑	↑	↑	↑	↓	↓	↓	↓	↓	↓

An upward pointing arrow denotes a significant increase in response compared to the response in RPMI + HS, a downwards pointing arrow denotes a significant decrease and a ‘dash’ denotes no significant change. See also Figure 1; Supplementary Figures S1-S12.

**FIGURE 2:**
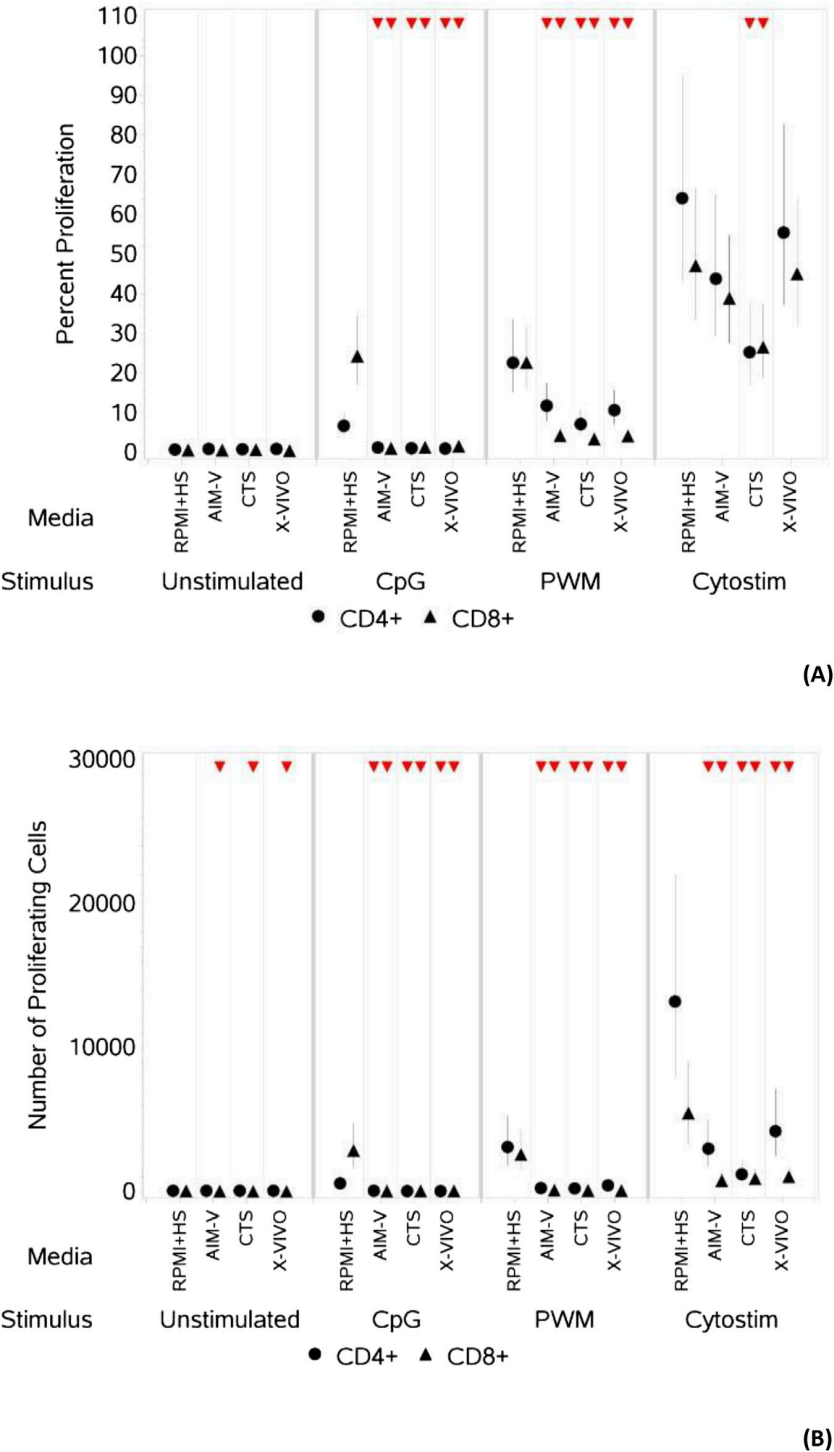
Impact of use of different media on percentage **(A)** and absolute number **(B)** of proliferating CD4^+^ and CD8^+^ T cells, with and without stimulation. PBMCs were isolated from healthy donors, stained with the proliferation dye eFluor 450^®^ and cultured in the absence (unstimulated) or presence of stimulation reagents (CpG-ODN plus IL-15, Pokeweed Mitogen (PWM) or Cytostim (CS)), in RPMI plus human serum, AIM-V, CTS OpTmizer T cell expansion SFM or X-VIVO 15 media. Proliferation was measured after 6 days by flow cytometry and Zombie Green viability dye was used along with anti-human CD3, CD4 and CD8 as detailed in the materials and methods section. Red triangles denote a significant decrease compared to RPMI + HS, and green triangles indicate a significant increase. Points and lines represent the mean and 95% confidence interval of the mean after the donor effect has been accounted for (eight donors, each with three measurements), respectively.

As summarised in Table 3 and shown in Figures 2A, B, for unstimulated cells there was no significant difference in percentage or number of proliferating CD4^+^ T cells across all four media. There was also no difference in the percentage of proliferating CD8^+^ T cells, there was however a small but significantly lower number of proliferating CD8^+^ T cells in the three serum-free media compared to RPMI + HS. There was also a significantly lower percentage and number of proliferating CD4^+^ and CD8^+^ T cells after stimulation with CpG + IL15 and PWM in all three serum-free media compared to RPMI + HS. With CS whilst this was again seen across all three serum-free media for the number of proliferating CD4^+^ and CD8^+^ T cells, there was only a significantly lower percentage of proliferating CD4^+^ and CD8+T cells in CTS media compared to RPMI + HS.

**TABLE 3:** Changes in the percentage and number of proliferating CD4^+^ and CD8^+^ T cells in different serum-free media compared to RPMI + HS, when unstimulated and stimulated.

Condition	Changes in the percentage and number of proliferating CD4^+^ and CD8^+^ cells											
	Percent proliferating cells						Number of proliferating cells					
	AIM-V CD4^+^ CD8^+^		CTS CD4^+^ CD8^+^		X-VIVO CD4^+^ CD8^+^		AIM-V CD4^+^ CD8^+^		CTS CD4^+^ CD8^+^		X-VIVO CD4^+^ CD8^+^	
Unstimulated	-	-	-	-	-	-	-	↓	-	↓	-	↓
CpG	↓	↓	↓	↓	↓	↓	↓	↓	↓	↓	↓	↓
PWM	↓	↓	↓	↓	↓	↓	↓	↓	↓	↓	↓	↓
CS	-	-	↓	↓	-	-	↓	↓	↓	↓	↓	↓

An upward pointing arrow denotes a significant increase in response compared to the response in RPMI + HS, a downwards pointing arrow denotes a significant decrease and a ‘dash’ denotes no significant change. See also Figure 2; Supplementary Figures S1-S12.

### 3.2 B cell viability and proliferation

**FIGURE 3:**
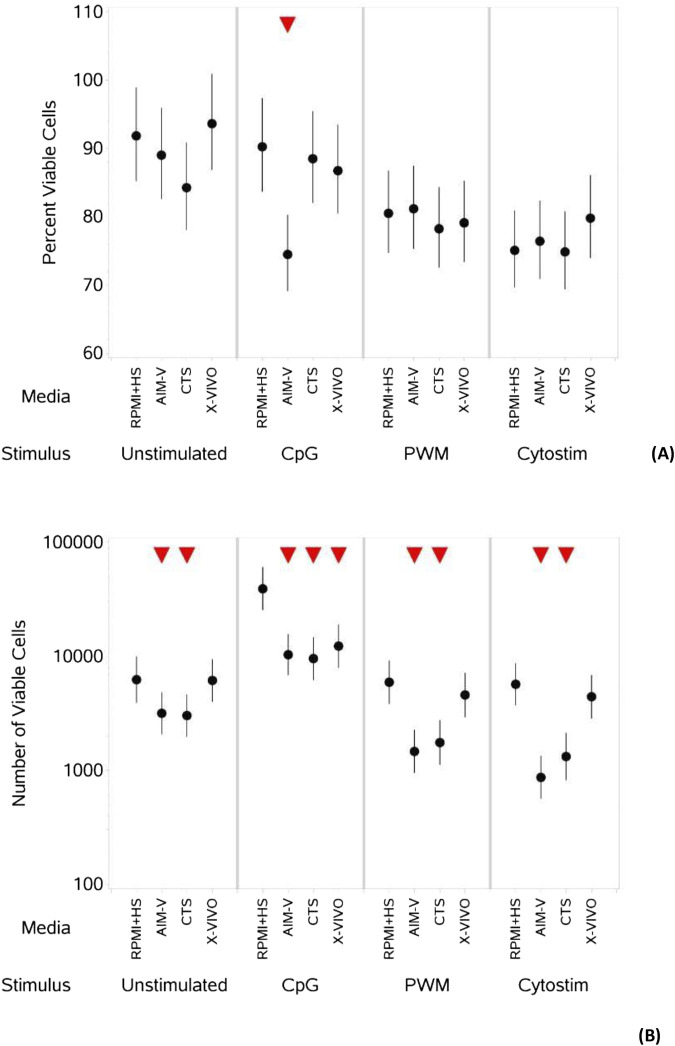
Impact of use of different media on percentage **(A)** and absolute number **(B)** of viable B cells, with and without stimulation. PBMCs were isolated from healthy donors and cultured in the absence (unstimulated) or presence of stimulation reagents (CpG-ODN plus IL-15, Pokeweed Mitogen (PWM) or Cytostim (CS)), in RPMI plus human serum, AIM-V, CTS OpTmizer T cell expansion SFM or X-VIVO 15 media. Viability was measured after 6 days by flow cytometry and Zombie Green viability dye was used along with anti-human CD19 as detailed in the materials and methods section. Red triangles denote a significant decrease compared to RPMI + HS, and green triangles indicate a significant increase. Points and lines represent the mean and 95% confidence interval of the mean after the donor effect has been accounted for (eight donors, each with three measurements), respectively.

The mean percentage of viable B cells remained above 75% in all four media (Figure 3), whether unstimulated or stimulated.

As summarised in Table 4, when comparing across media (Figure 3A) there was no significant difference in the percentage of viable B cells stimulated with PWM and CS, but with CpG + IL15 there was a small but significantly lower percentage of viable B cells in AIM-V media compared to RPMI + HS. Without stimulation there was a small but significantly lower number of viable B cells (Figure 3B) in AIM-V and CTS compared to RPMI + HS. For stimulation with PWM and CS this lower number was also seen in AIM-Vand CTS compared to RPMI + HS, and with CpG + IL15 in all three serum-free media.

**TABLE 4:** Changes in the percentage and number of viable B cells in different serum-free media compared to RPMI + HS, when unstimulated and stimulated.

Condition	Changes in the percentage and number of viable B Cells					
	Percent viable cells			Number of viable cells		
	AIM-V	CTS	X-VIVO	AIM-V	CTS	X-VIVO
Unstimulated	-	-	-	↓	↓	-
CpG	↓	-	-	↓	↓	↓
PWM	-	-	-	↓	↓	-
CS	-	-	-	↓	↓	-

An upward pointing arrow denotes a significant increase in response compared to the response in RPMI + HS, a downwards pointing arrow denotes a significant decrease and a ‘dash’ denotes no significant change. See also Figure 3; Supplementary Figures S1-S12.

**FIGURE 4:**
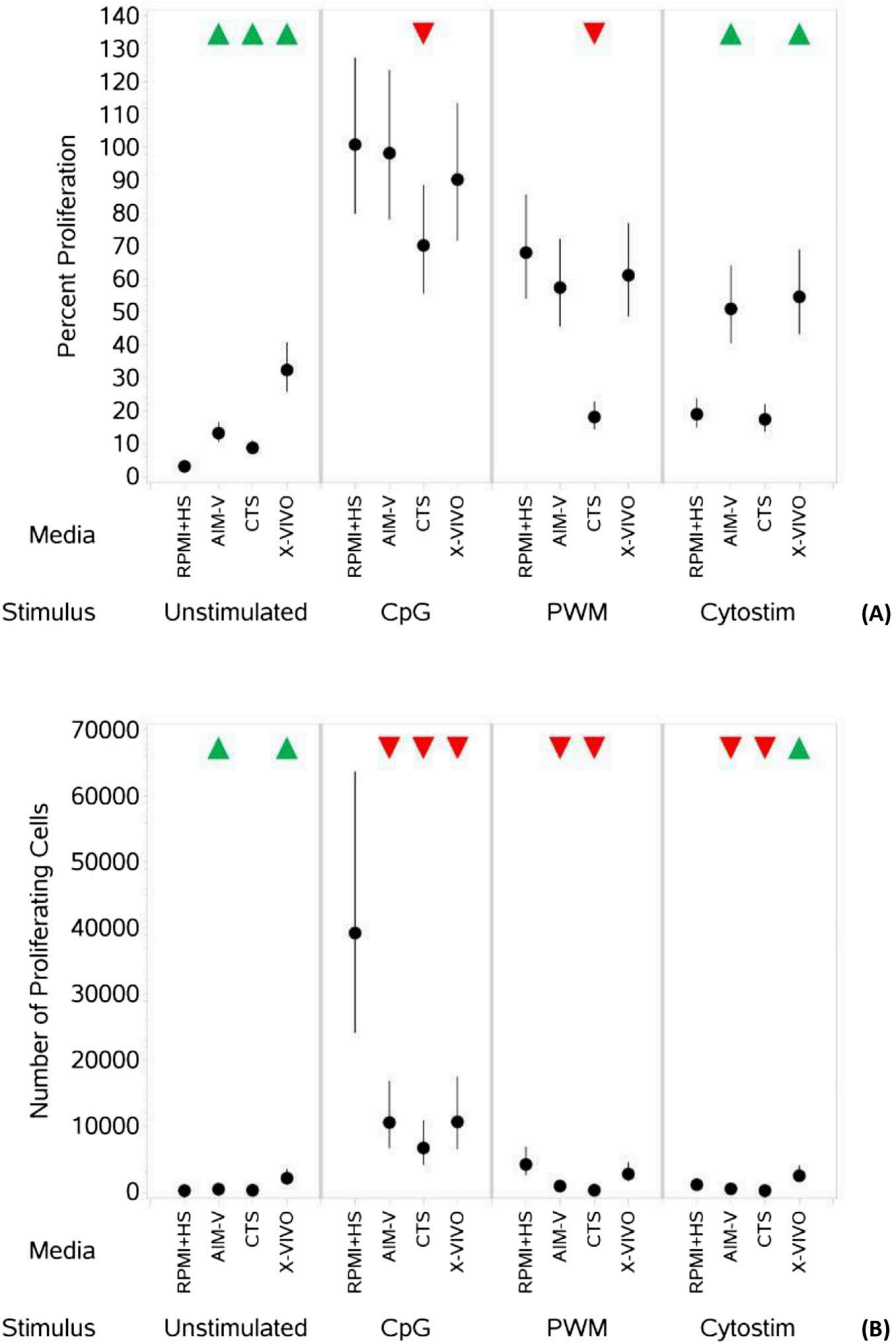
Impact of use of different media on percentage **(A)** and absolute number **(B)** of proliferating B cells, with and without stimulation. PBMCs were isolated from healthy donors, stained with the proliferation dye eFluor 450^®^ and cultured in the absence (unstimulated) or presence of stimulation reagents (CpG-ODN plus IL-15, Pokeweed Mitogen (PWM) or Cytostim (CS)), in RPMI plus human serum, AIM-V, CTS OpTmizer T cell expansion SFM or X-VIVO 15 media. Proliferation was measured after 6 days by flow cytometry and Zombie Green viability dye was used along with anti-human CD19 as detailed in the materials and methods section. Red triangles denote a significant decrease compared to RPMI + HS, and green triangles indicate a significant increase. Points and lines represent the mean and 95% confidence interval of the mean after the donor effect has been accounted for (eight donors, each with three measurements), respectively.

As summarized in Table 5, when unstimulated, there was an increase in the percentage of proliferating B cells (Figure 4A) in all three serum-free media compared to RPMI + HS, with X-VIVO media supporting the highest percentage proliferation. A similar result is seen for the numbers of proliferating B cells (Figure 4B), though in CTS this was not significantly different. When stimulated with CpG + IL15 and PWM the percentage of proliferating B cells was significantly lower in CTS media compared to RPMI + HS, but not in AIM-V and X-VIVO. With CS this value significantly increased compared to RPMI + HS in AIM-V and X-VIVO media, but not in CTS. With stimulation numbers were lower in the three serum-free media than RPMI + HS when stimulated with CpG + IL15, lower in AIM-Vand CTS when stimulated with PWM and CS but increased with CS in X-VIVO media, compared to RPMI + HS.

**TABLE 5:** Changes in the percentage and number of proliferating B cells in different serum-free media compared to RPMI + HS, when unstimulated and stimulated.

Condition	Changes in the percentage and number of proliferating B Cells					
	Percent proliferating B Cells			Number of proliferating B Cells		
	AIM-V	CTS	X-VIVO	AIM-V	CTS	X-VIVO
Unstimulated	↑	↑	↑	↑	-	↑
CpG	-	↓	-	↓	↓	↓
PWM	-	↓	-	↓	↓	-
CS	↑	-	↑	↓	↓	↑

An upward pointing arrow denotes a significant increase in response compared to the response in RPMI + HS, a downwards pointing arrow denotes a significant decrease and a ‘dash’ denotes no significant change. See also Figure 4; Supplementary Figures S1-S12.

### 3.3 IgG levels in the cell culture supernatants

**FIGURE 5:**
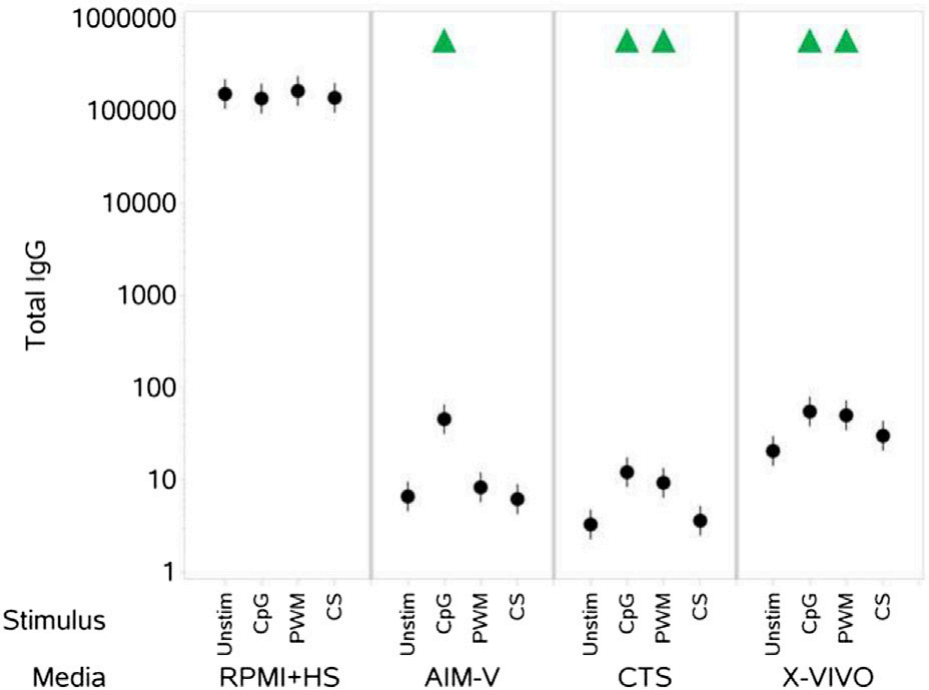
Impact of stimuli in different media on total IgG levels (ng per mL). PBMCs were isolated from healthy donors, stained and cultured in the absence (unstimulated) or presence of stimulation reagents (CpG-ODN plus IL-15, Pokeweed Mitogen (PWM) or Cytostim (CS)), in RPMI plus human serum, AIM-V, CTS OpTmizer T cell expansion SFM or X-VIVO 15 media. Total IgG levels in the cell culture supernatants were measured after 6 days by ELISA as detailed in the materials and methods section. Red triangles denote a significant decrease compared to the unstimulated control, and green triangles indicate a significant increase. Points and lines represent the mean and 95% confidence interval of the mean after the donor effect has been accounted for (eight donors, each with three measurements), respectively. Where values were below or above, the lower and upper limits of quantification respectively, they were substituted with the relevant limit value.

CpG + IL-15 stimulation of PBMCs cultured in all three serum-free media resulted in a measurable increase in levels of IgG measured in the cell culture supernatant (Figure 5). PWM also induced an increase in IgG levels in CTS and X-VIVO media. A stimulation-evoked increase in IgG levels was not evident for PBMCs cultured in RPMI + HS and the levels were many orders of magnitude higher than those in the serum-free media.

## 4 Discussion

Developed in 1966, by Moore et al. (Moore et al., 1967) and named for the Roswell Memorial Institute where it was developed, RPMI became the first serum-free medium for the expansion of PBMCs. Simultaneously, researchers established a traditional cell culture protocol using RPMI supplemented with foetal bovine serum (FBS), which became the most used medium for PBMC expansion. However, early observations revealed that the serum supplement significantly influenced experimental results due to poorly defined components (e.g., growth factors, antibodies, and other immunologically active substances) that varied in concentration between batches. This variability hindered standardised and reproducible cell preparation and assay evaluation, as noted by Yssel et al. (Yssel et al., 1984) and Polet and Spieker-Polet (Polet and Spieker-Polet, 1975). Despite efforts in the late 1970s to eliminate serum from cell culture media due to concerns about reproducibility, reliability, safety relevance, and ethical issues, RPMI supplemented with FBS has continued to be widely used for PBMC culture. It is only in recent years that these issues have regained significant attention (van der Valk, 2022; van der Valk et al., 2018). Additionally, the discussion around use of serum in culture media has further expanded to consider use of animal-derived materials more widely (Duarte et al., 2023) and drive the development of different chemically defined media for different cell lines and applications.

Recognising the issues associated with use of FBS and to maximise the human relevance of the PBMC-based assays being used to assess the impact of exogenous materials on the human immune system, we chose to use RPMI supplemented with human serum, and this is media used in the work captured in Cochrane et al. (2024). As described in the introduction, however, this did come with some challenges, including an inability to measure induced levels of immunoglobulins due to high background levels in human serum.

In efforts to address this specific concern, the work described was undertaken to determine if three commercially available, chemically defined and serum-free media could be used to culture PBMCs *in vitro* and enable measurement of changes in IgG levels with stimuli, with a view to assessing any impact of exogenous materials on these responses.

In this study we assessed AIM-V, CTS and X-VIVO media and provide details of the key components of these media, indicated cell types/applications and classifications in Table 1. With regard to the history of these media, in the late 1980s and early 1990s, proprietary cell culture media for T cell expansion emerged through modifications of both IMDM and DMEM: F12. Extensive adaptations to DMEM: F12 led to the creation of GIBCO AIM-V in 1987, the first chemically defined serum-free medium for the proliferation of T-cells and dendritic cells. Meanwhile, modifications to IMDM gave rise to the X-VIVO series of hematopoietic media and later, the third medium, CTS OpTmizer SFM was developed as a more robust medium for high-density T cell expansion.

AIM-V medium is supplemented with purified human albumin, transferrin, insulin, and a proprietary blend of purified factors to promote cell and tissue growth. Lonza X-VIVO-15 medium contains human albumin, recombinant human insulin, and pasteurised human transferrin. CTS OpTmizer T Cell Expansion medium and supplement contains HSA. It also contains N-acetyl-cysteine (NAC), increased calcium and magnesium, and sodium pyruvate (a frequently included additive in cell culture media) as an additional carbon source alongside glucose, providing antioxidant and anti-inflammatory properties (Sato et al., 2009) and https://assets.fishersci.com/TFS-Assets/CMD/brochures/Serum-Free-Optimizer.pdf).

Insulin has cell signaling functions and promotes the uptake of glucose and amino acids. Transferrin is a universal source of iron for cells that is non-toxic carrier of iron and reduces the generation of toxic free-radicals and peroxide. Glutamine is one of the most readily available amino acids for use as an energy source and it is a major source of energy for many rapidly dividing cell types *in vitro*. AIM-V is used for adoptive immunotherapy (lymphokine-activated killer (LAK) cells) clinical trials. In addition, the medium is reported to be useful in cultivating monocytes, dendritic cells, T cells, hybridomas, PBMCs, fibroblasts, macrophages, myeloma cells, lymphocytes. In AIM-V medium, additive levels are not explicitly specified and protected in FDA drug master file.

Each of the serum-free media tested (AIM-V, CTS, and X-VIVO) supported good levels of viable and proliferating T cells and B cells over the 6 days of culture, with only a few, small differences (slightly lower number of viable CD8^+^ T cells in the three serum-free media, slightly lower numbers of viable B cells in AIM-V and CTS media but higher (both percentage and number) proliferating B cells in AIM-Vand X-VIVO) across the media when there was no stimulation. They also enabled detection of a stimulation-evoked increase in IgG levels.

In contrast, although RPMI + HS also supported good levels of viable and proliferating T and B cells over the 6 days, a stimulation-evoked increase in IgG levels was not evident due to the already high pre-existing IgG levels in the human serum, essentially masking any stimulation induced IgG production.

The T and B cell viability and proliferation profiles in response to the selected concentrations of the three stimuli used confirm the data reported in Cochrane et al. (2024), when cultured in RPMI + HS. In brief, CS was the most potent stimulator of T cells (both CD4^+^ and CD8^+^) increasing viable cell numbers (though with a small reduction in percentage viable cells) and cell proliferation (both percentage and absolute numbers). Whilst it may seem contradictory for there to be a reduction (though small) in percentage viable cells when there is an increase in absolute viable cell numbers, this is possible and illustrates (as discussed in Cochrane et al., 2024) the importance of measuring both percentage viability and absolute viable cell numbers. These measurements, together with equivalent proliferation data provide an insight into the impact of stimuli and identify activation-induced death. The decrease in percentage viability observed, despite an increase in absolute viable cell number and proliferation upon stimulation, indicates that there may be some underlying cell death impacting the percentage of viable cells, with the trade-off between proliferation and cell death specific to the stimulus, its concentration, and cell population.

CpG + IL15 was the most potent stimulator of B cells increasing the total number of viable B cells and both the percentage and total number of proliferating B cells, which is as expected given that human memory B cells have been reported to exhibit vigorous *in vitro* proliferation when exposed to a combination of IL-15 and CpG DNA (Bernasconi et al., 2002). PWM however failed to increase the absolute number or percentage of viable B cells compared to unstimulated cells and whilst it increased the percentage proliferating B cells it was to a lesser extent than CpG + IL15. Additionally, when the number of proliferating cells was assessed, there was no significant change in two media and only relatively small increases from a low baseline number in the others. This again reflects the trade-off between proliferation and viability as described in the previous paragraph and different modes of action of CpG + IL15 and PWM. CpG + IL15 and PWM also increased the percentage and number of proliferating CD4^+^ and CD8^+^ T cells but to a lesser degree than CS. PWM was also more stimulating than CpG + IL15 for CD4^+^ T cells, reflecting the different mechanisms involved with PWM engaging multiple cell types within the PBMC sample. For example, TLR activation of dendritic cells and NK cells can stimulate bystander T cell activation through the release of IFNαβ and IFNγ (Kamath et al., 2005). There may also be an unspecified component in serum that enhances CpG-uptake into an immune cell type, which subsequently supports CD8^+^ T cell differentiation in a similar manner to the requirement for LPS-binding protein in serum regulating the response of monocytes to LPS (Gallay et al., 1994). Alternatively, Fc-receptor tonic signalling through serum immunoglobulins might lower the activation threshold in certain immune cell types (e.g., monocytes or NK cells) so that CpG + IL-15 is sufficient to trigger their activation, and subsequent bystander activation of CD8^+^ T cells. In addition, IL-15 has been reported to activate CD8^+^ T cells (Anthony and Schluns, 2015) and NK cells (Ma et al., 2022). In the current experimental conditions, the presence of human serum may have allowed an IL-15-induced response to have been revealed.

Use of the different media also impacted the viability and proliferation responses of T and B cells to different stimuli. In general, the differences were small across all the different media for T cell responses, but of note was the observation that CTS medium supported a significantly lower percentage of proliferating T cells after stimulation with CS when compared to RPMI + HS and lowest of the all the media, with correspondingly low numbers of proliferating T cells. B cell viability and proliferation measurements after stimulation were, overall, highest in X-VIVO media of all the serum-free medium and lowest in CTS media. Stimulation with CpG + IL-15 increased levels of IgG in PBMCs culture in all the serum-free media. However, a PWM induced increase in IgG levels was only significant in X-VIVO and CTS media, and greater in the former.

X-VIVO medium has previously been demonstrated to better support T cell expansion over many days of culture compared to AIM-V medium (Medvec et al., 2018; Carlens et al., 2000), and Martinuzzi et al. reported that PBMCs stimulated in AIM-V medium yielded higher antigen specific signals in IFNg-ELISPOTs than those cultured in RPMI + HS (Martinuzzi et al., 2008). As PWM evoked B cell activation is dependent on T cells (Hammarstrom et al., 1979), the sustained T cell support provided by the X-VIVO media, may lead to an enhancement of B cell IgG production. Alternatively, the higher basal level of B cell proliferation evident in the X-VIVO medium compared to the other test media suggests components within this medium may have a direct stimulatory impact on B cells. Serum-free media formulations could also differentially impact T cell phenotype and subsequently their ability to support B cell IgG production in response to the T cell - dependent stimulus PWM. For example, Sato et al. reported that CTS medium contains NAC, which is a compound that scavenges reactive oxygen intermediates but that has also been shown to increase intracellular glutathione levels and to affect differentially the cytokine-stimulated proliferation of T cells (Sato et al., 2009). The lack of any insulin or transferrin in the CTS media may also be explain why this media performed less well than X-VIVO and Smith et al. reported that use of a xeno-free supplement CTS Immune Cell SR (Serum Replacement), which is indicated by the manufacturer to contain human and recombinant proteins, supported better expansion of T cells (Smith et al., 2015).

For this study we did not explore other readouts such as cytokine production or activation markers, and only a limited set of stimuli and as such the potential influence of serum-free media on these would need exploring. From the available literature there are signs that other factors in serum, that are not currently in some serum-free media could be required for responses to some materials. For example, Slunt et al. report that *in vitro* T-cell responses to the dermatophyte fungus *Trichophyton tonsurans* were inhibited by the serum-free medium AIM-V, with inhibition seen equally with cells from individuals with delayed and immediate hypersensitivity (Slunt et al., 1997).

Human serum is a complex material (Psychogios et al., 2011) and does not simply serve as a nutrient source but plays multiple roles that all need consideration in the context of *in vitro* cell culture. For example, sometimes 2-mercaptoethanol (2- ME) is added to lymphocyte cultures to enhance responses, as has been found to be the case for murine cells. The impact on human cells is, however, variable and dependent upon the cell type and culture conditions, with the addition of 2-ME providing more enhancement in serum-free media (Click, 2014). To avoid any interference with PBMC responses to different exogenous materials, some of which may act via oxidative stress, we did not include 2-ME in the culture conditions. We also took the findings of Click (2014) into account and as we were supplementing RPMI with HS the addition of 2-ME was concluded to likely be of limited value given the potential interference it could introduce. It could also be hypothesised, based on the findings of Click (2014), that serum endows some oxidative stress resilience, which is lost in serum-free media and attempts to replace this with chemicals (e.g., with NAC in CTS) may introduce unexpected effects. The interaction of exogenous materials with serum is also an important consideration and understanding how this reflects human exposure and is therefore accurately replicated *in vitro*. The presence of human HSA in each of three media (Table 1) will, to some extent, cover key aspects such as the binding and release of materials, reflecting its role as a critical transport molecule, and HSA can also provide some oxidative stress protection through scavenging reactive oxygen species (Mishra and Heath, 2021).

There is therefore work still to be done to create a fully defined physiologically relevant medium for *in vitro* PBMC assays for assessing exogenous materials, that balances supporting cellular function and replicating ‘in human’ binding and release of such materials, with minimising any interference with modes of action. This would also address issues such as variability in composition and ethical concerns in some regions associated with using human sera (Jacobs et al., 2023). Use of statistical design, such as that utilised by Jeon et al. to develop serum-free media to support T lymphocyte expansion (Jeon et al., 2010), could be an efficient approach for this. An animal-free defined media has been developed and used for the 2D and 3D culture of a wide range of cell lines (normal and cancer) from different tissues (though not yet immune cells) to study proliferation, dose-response testing and migration that could also be investigated (Rafnsdottir et al., 2023; Weber et al., 2024).

In conclusion, the serum-free media formulations tested in this study offer defined systems for measuring B cell IgG responses, *in vitro*, in either a ‘T cell-independent’ (CpG + IL-15) or ‘T cell-dependent’ (PWM or CS) manner and for assessing B cell proliferation, particularly in response to a ‘T cell-independent’ stimulus. It is noted however that some characteristics and features endowed by serum appear to be missing when using such media. Therefore, there is further work to be done to optimise animal-free, chemically defined, culture conditions for PBMC based assays for inclusion in tiered safety assessments. For more discussion on these assays and their potential advantages and disadvantages with regards to their use in risk assessment, we refer readers to Cochrane et al. (2024). As described in Cochrane et al. (2024), RPMI + HS can be used successfully for many readouts to enable an *ab initio* assessment of the impact of exogenous materials on three major immune cell subsets, which is illustrated by initial profiling of the impact of curcumin as a case study material in that paper. Based on the results of this study, the use of RPMI + HS as described in Cochrane et al. (2024) could be combined with use of AIM-V and X-VIVO serum-free media for B-cell cultures with T cell independent and T cell dependent stimuli respectively, to also enable assessment of changes in IgG production.

## Data Availability

The raw data supporting the conclusions of this article will be made available by the authors, without undue reservation.
