# Supplementary figures and images for: Use of serum-free media for peripheral blood mononuclear cell culture and the impact on T and B cell readouts
Source: Front Toxicol. 2024 Nov 5;6:1462688. doi: 10.3389/ftox.2024.1462688 (PMC11573784; doi:10.3389/ftox.2024.1462688)

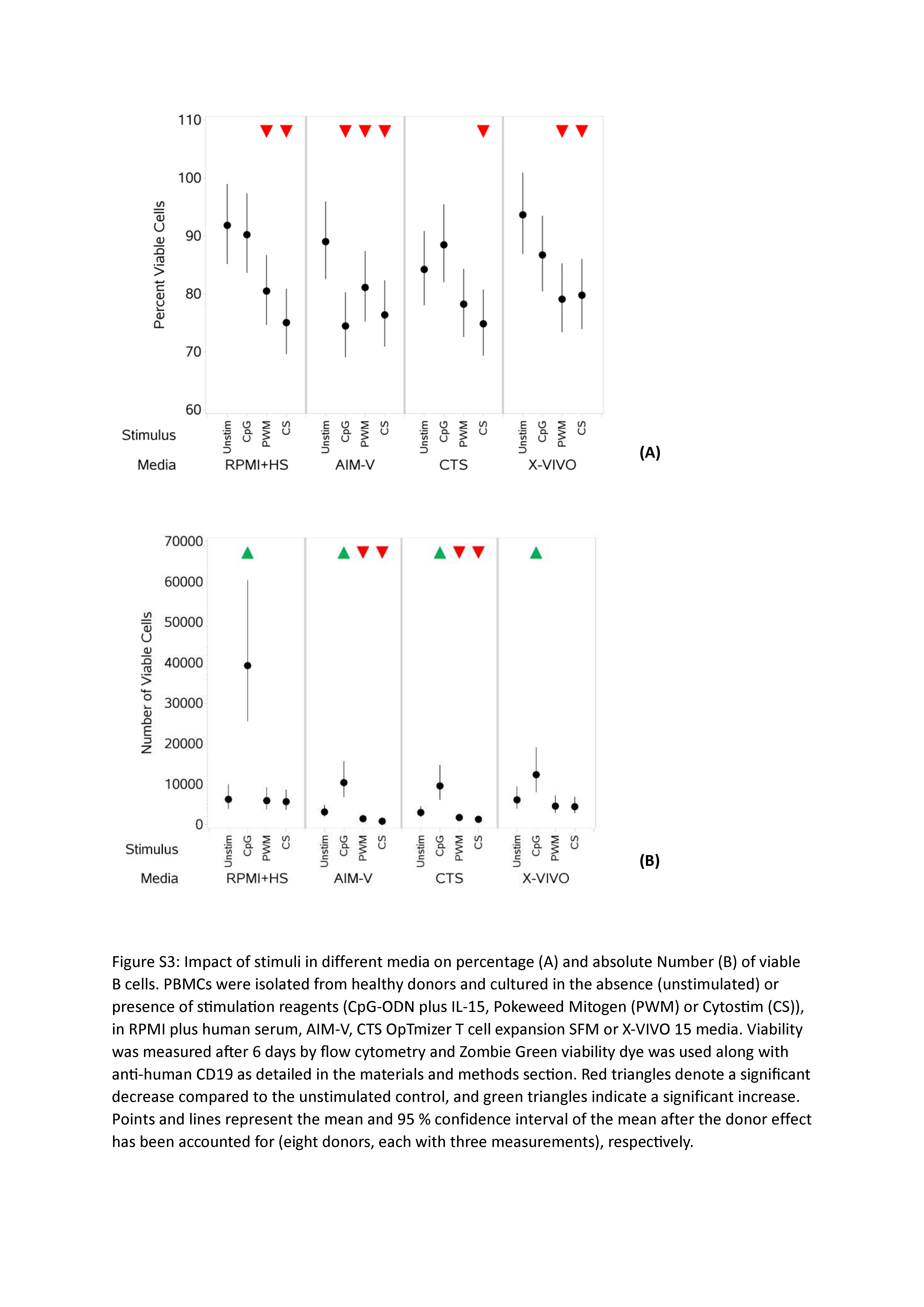

Supplement: Supplementary file 1 [file Image3.tiff]

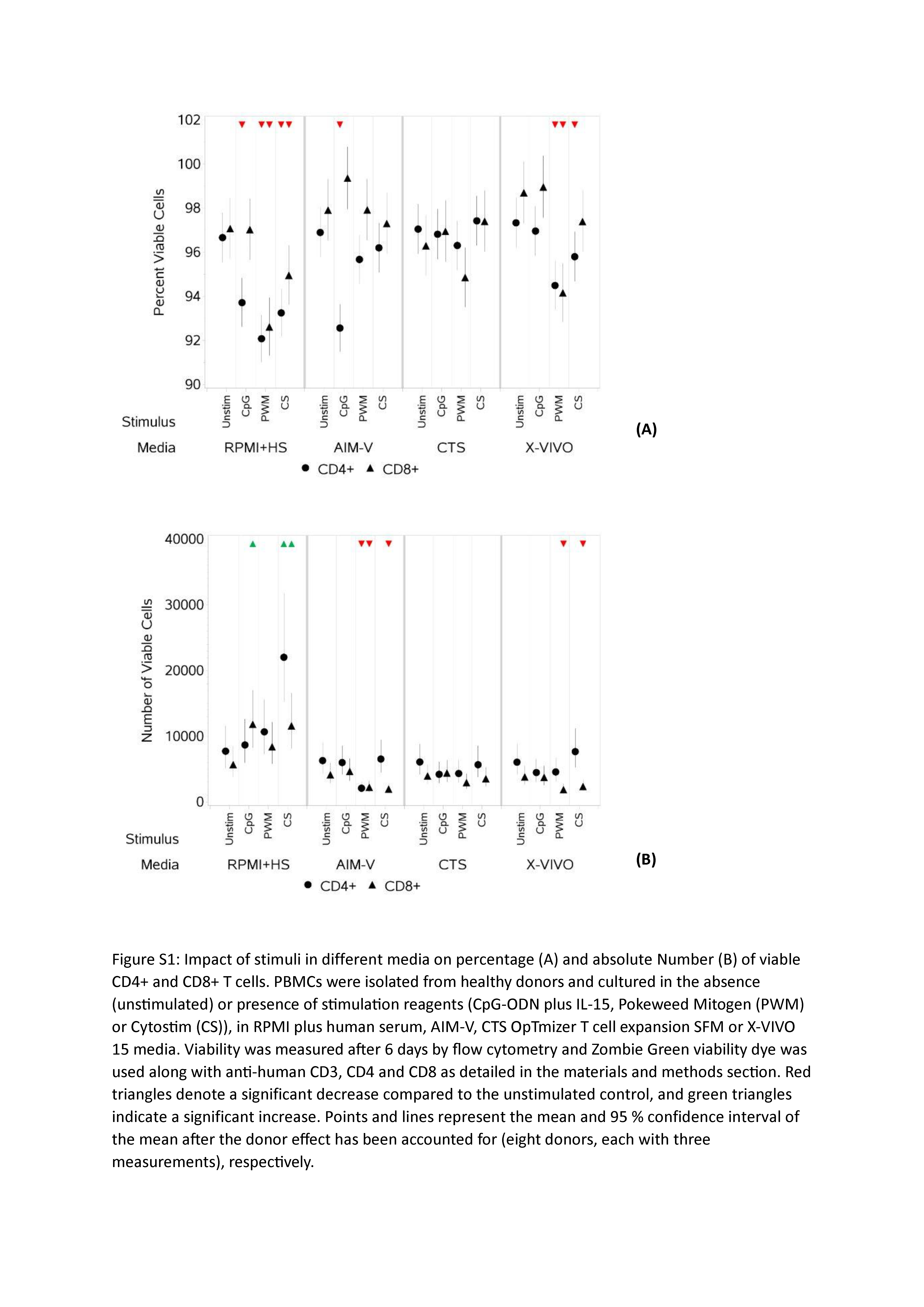

Supplement: Supplementary file 2 [file Image1.tiff]

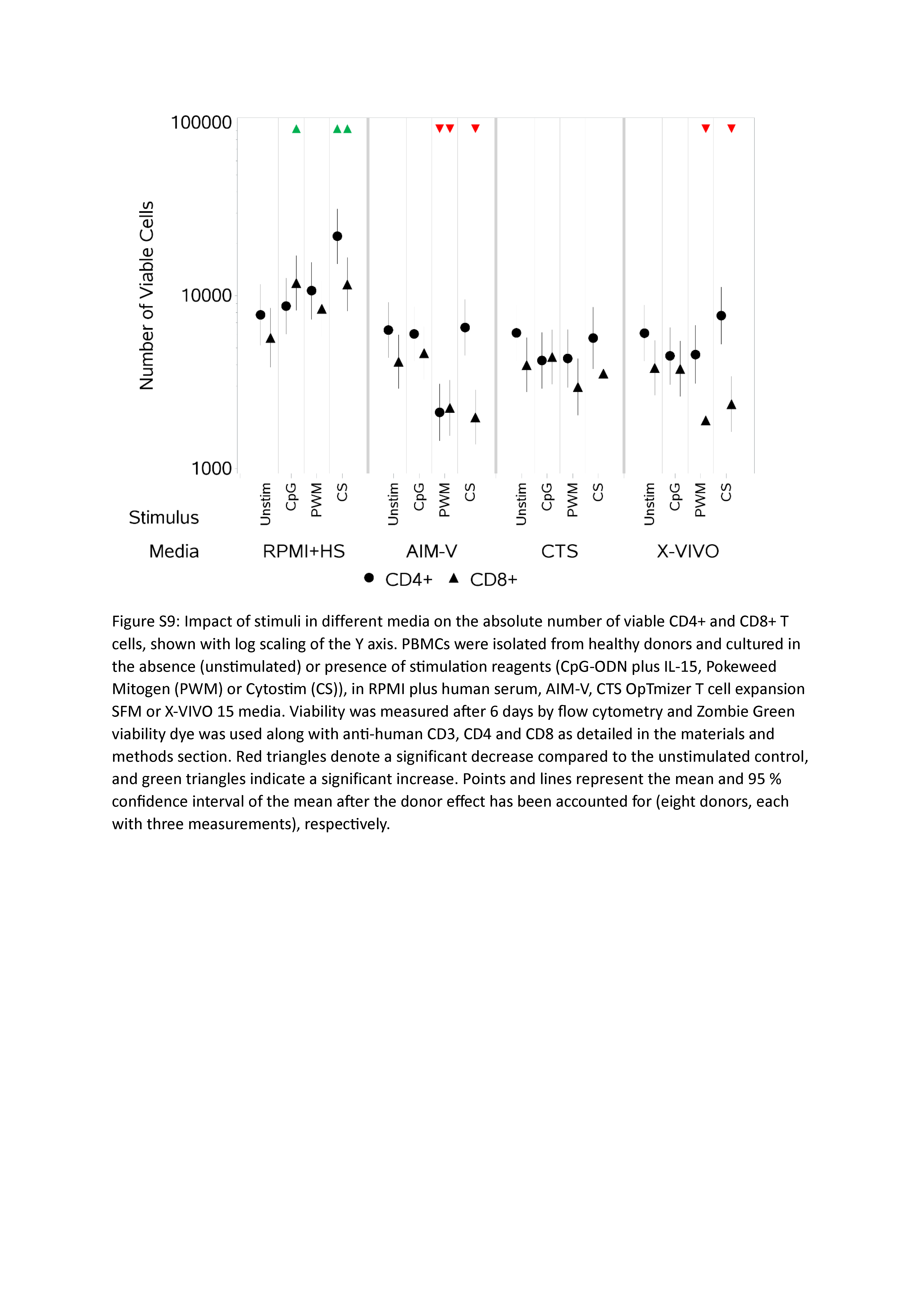

Supplement: Supplementary file 3 [file Image9.tiff]

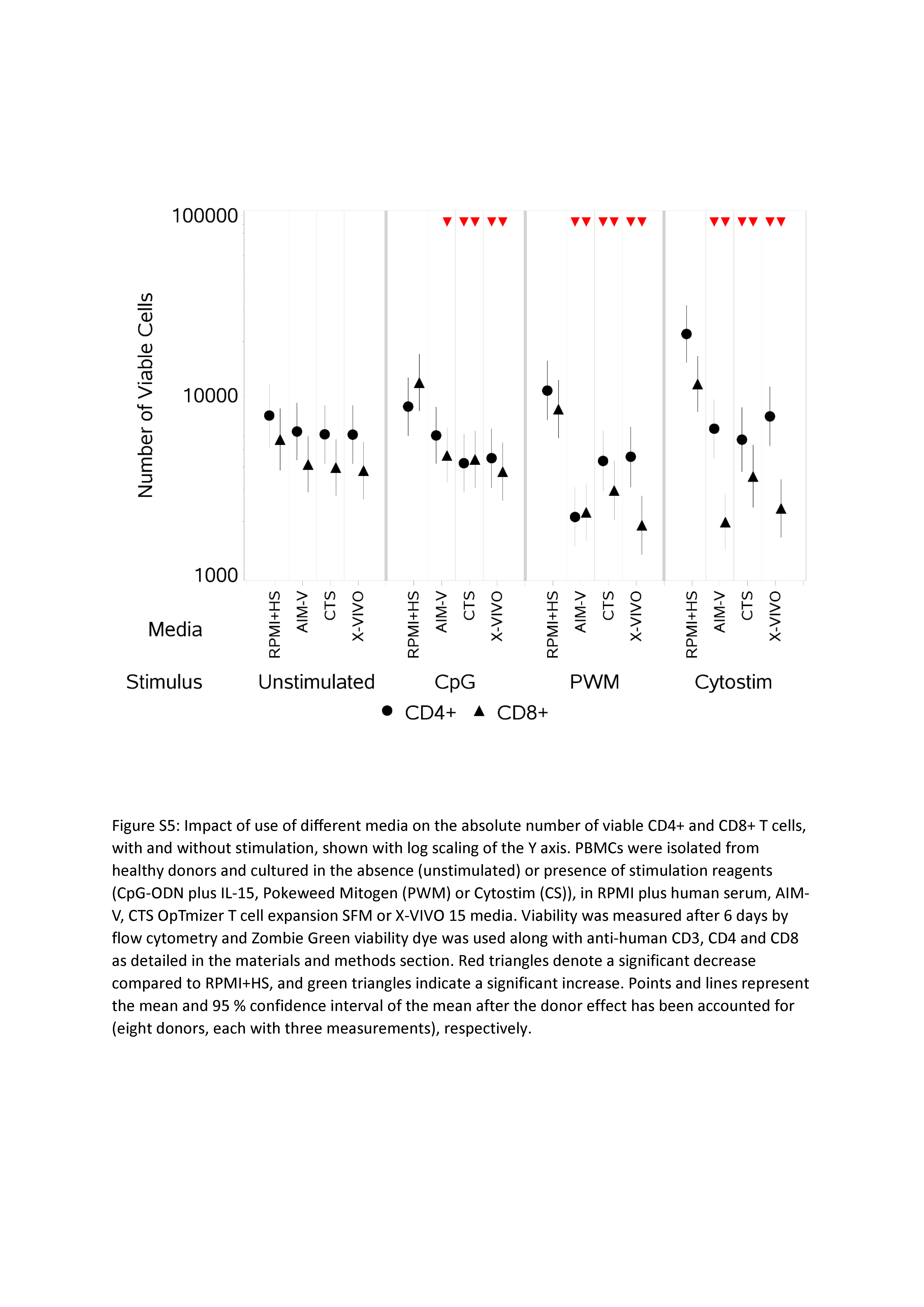

Supplement: Supplementary file 4 [file Image5.tiff]

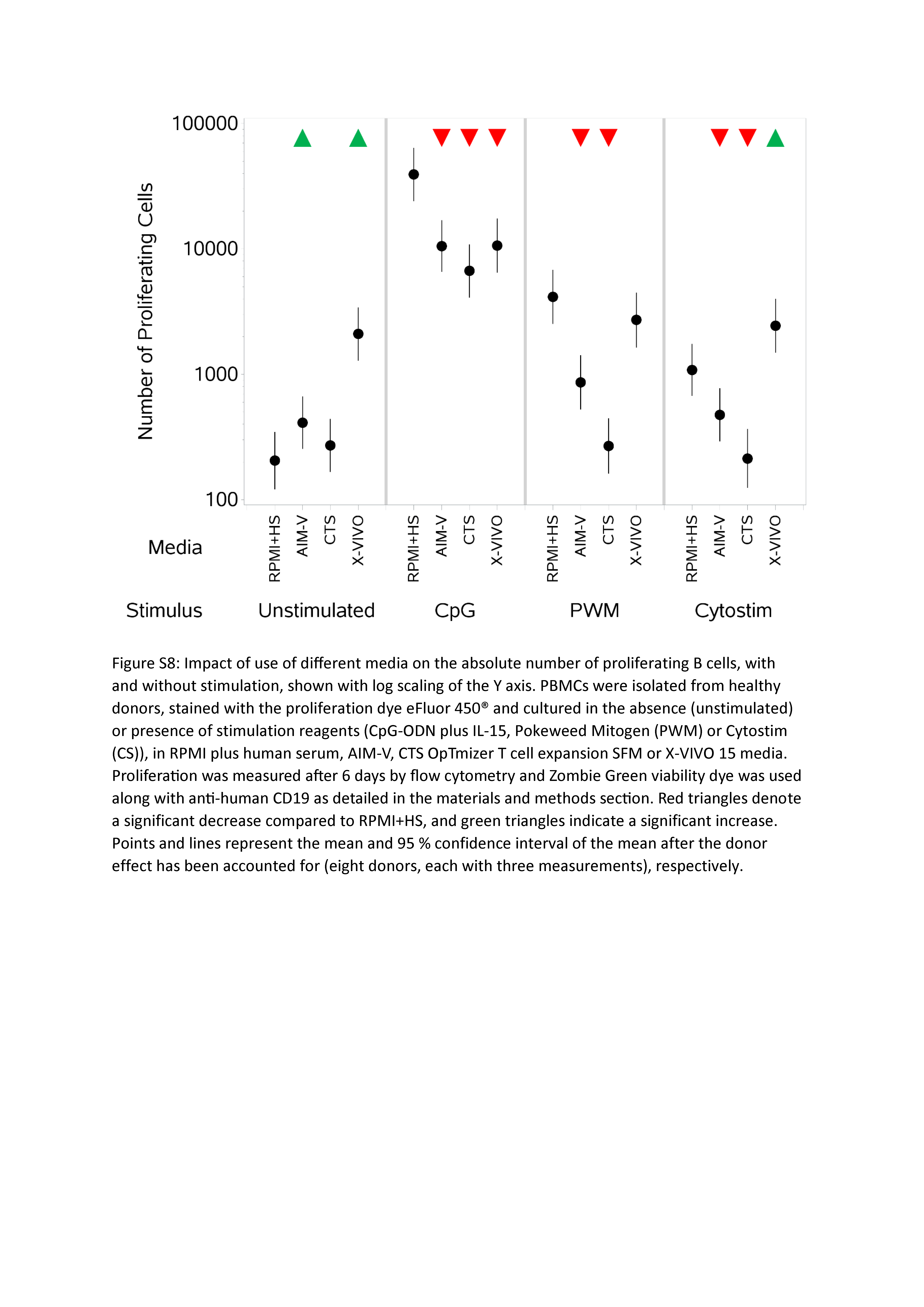

Supplement: Supplementary file 5 [file Image8.tiff]

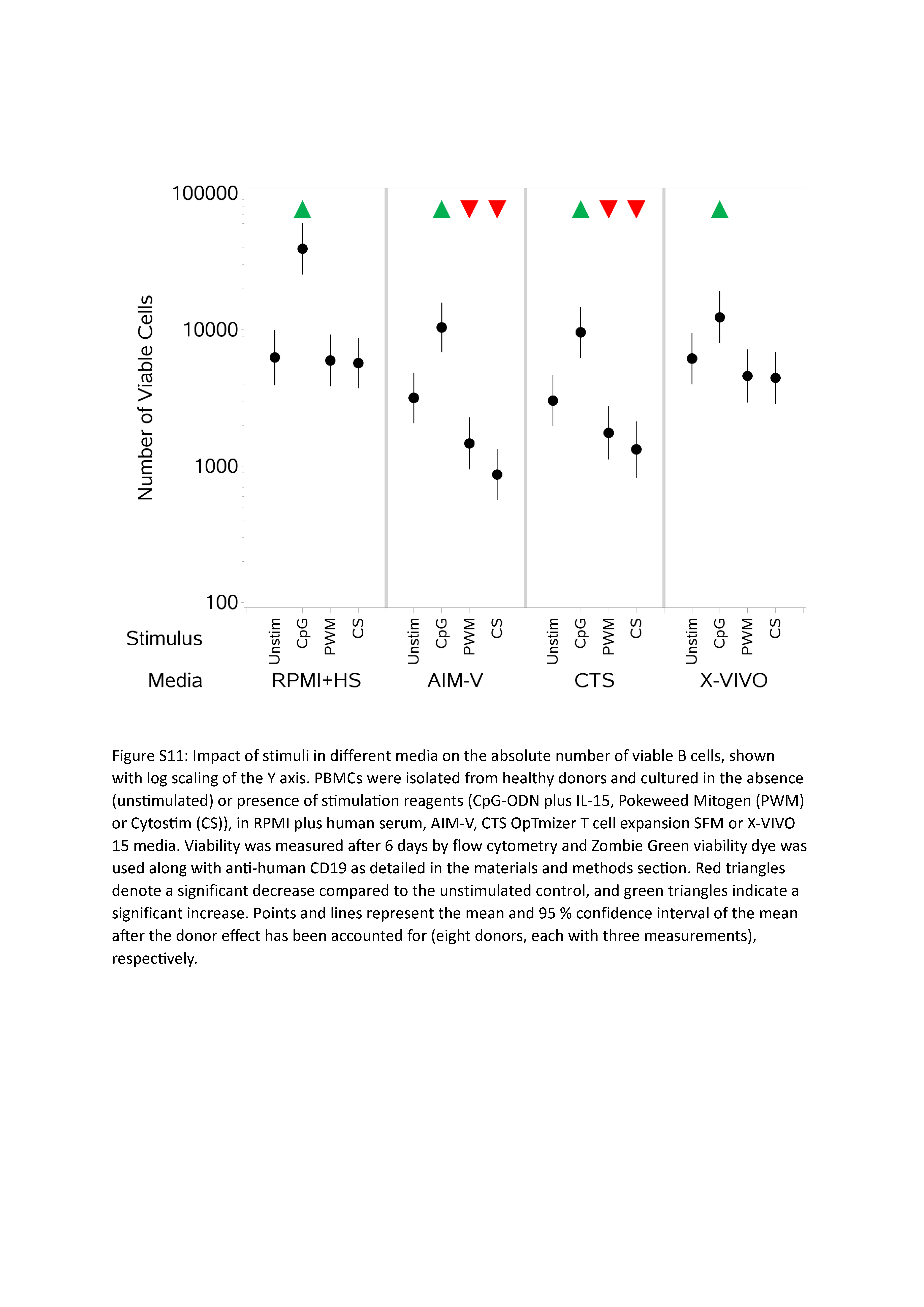

Supplement: Supplementary file 6 [file Image11.tiff]

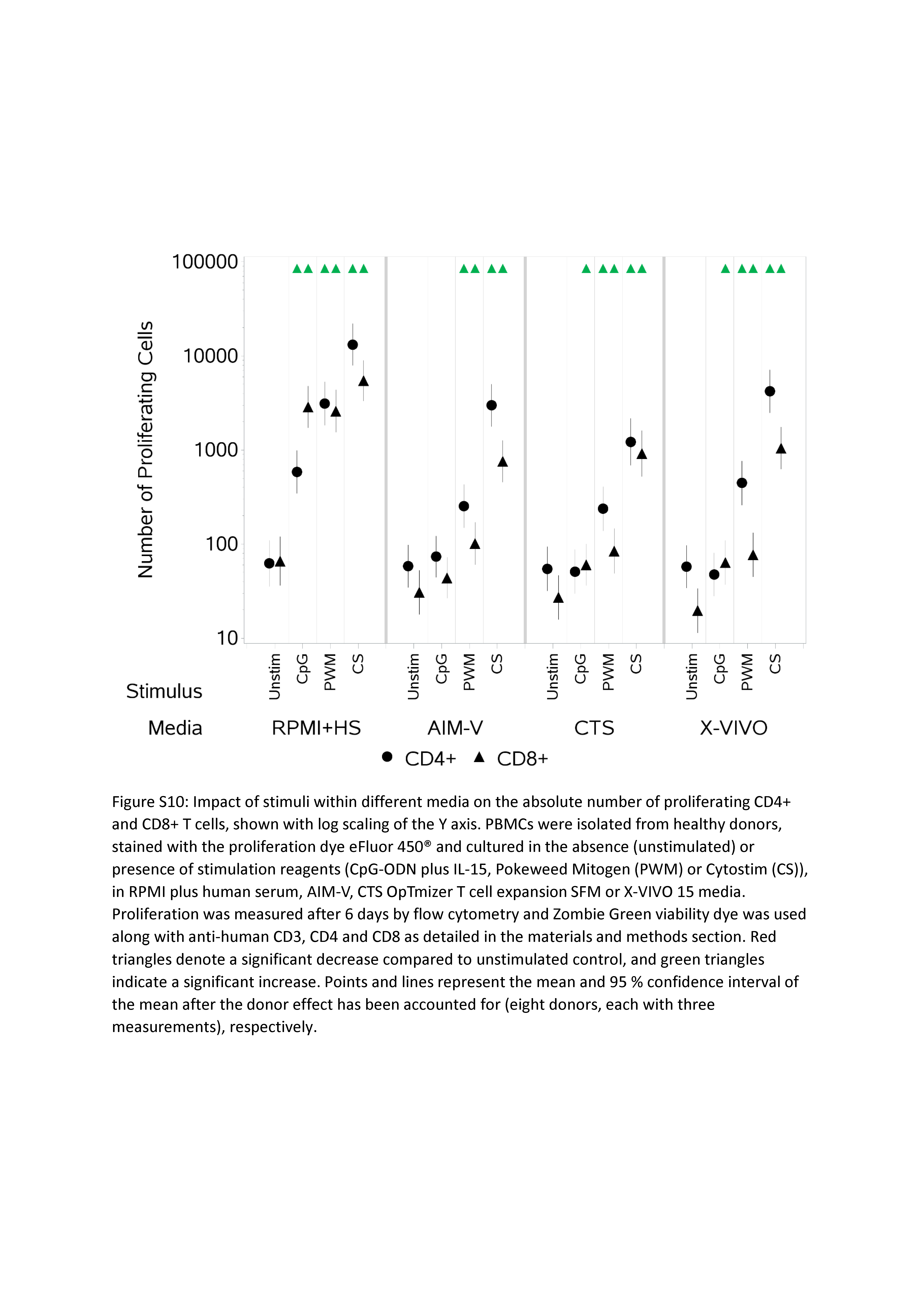

Supplement: Supplementary file 7 [file Image10.tiff]

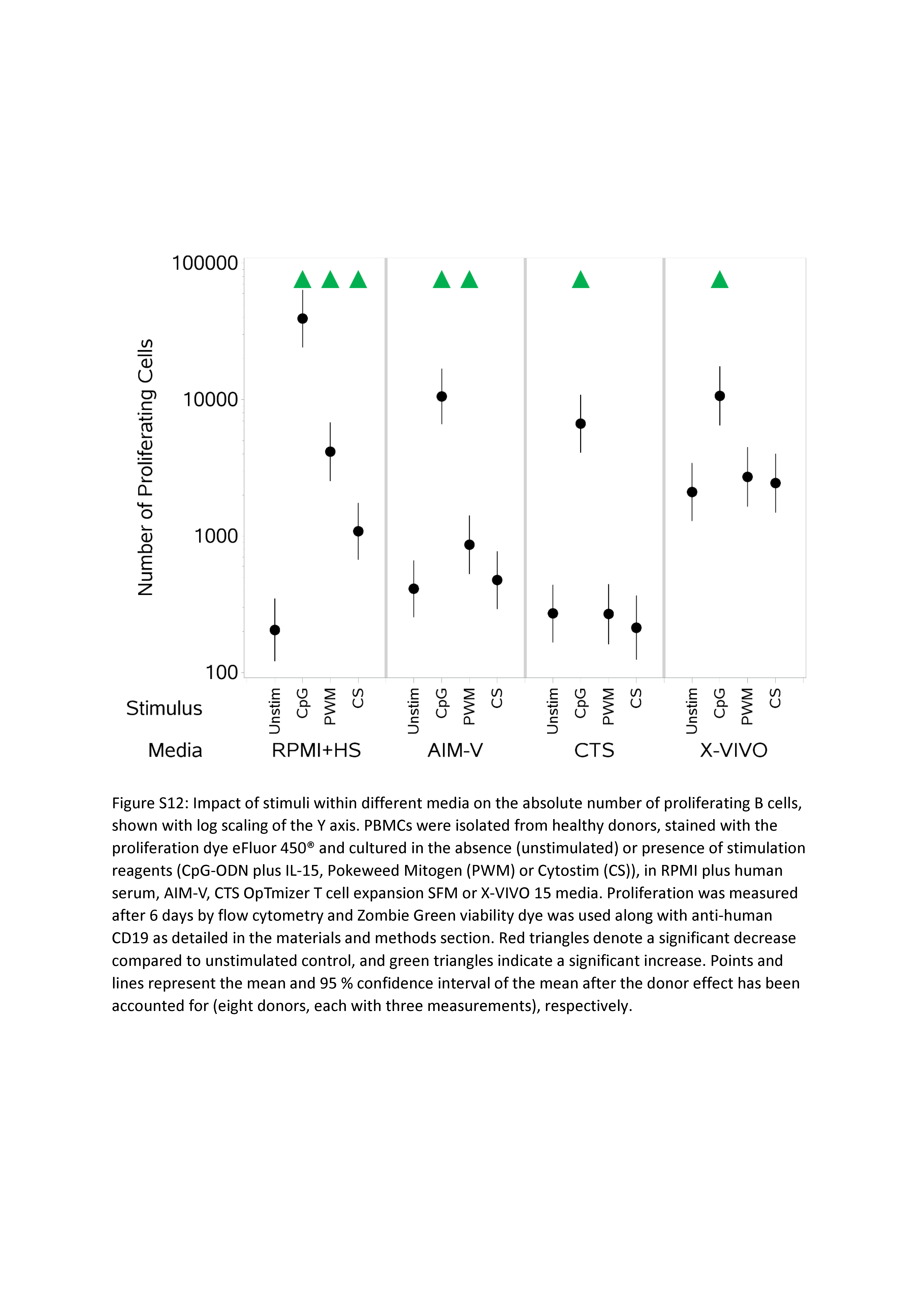

Supplement: Supplementary file 8 [file Image12.tiff]

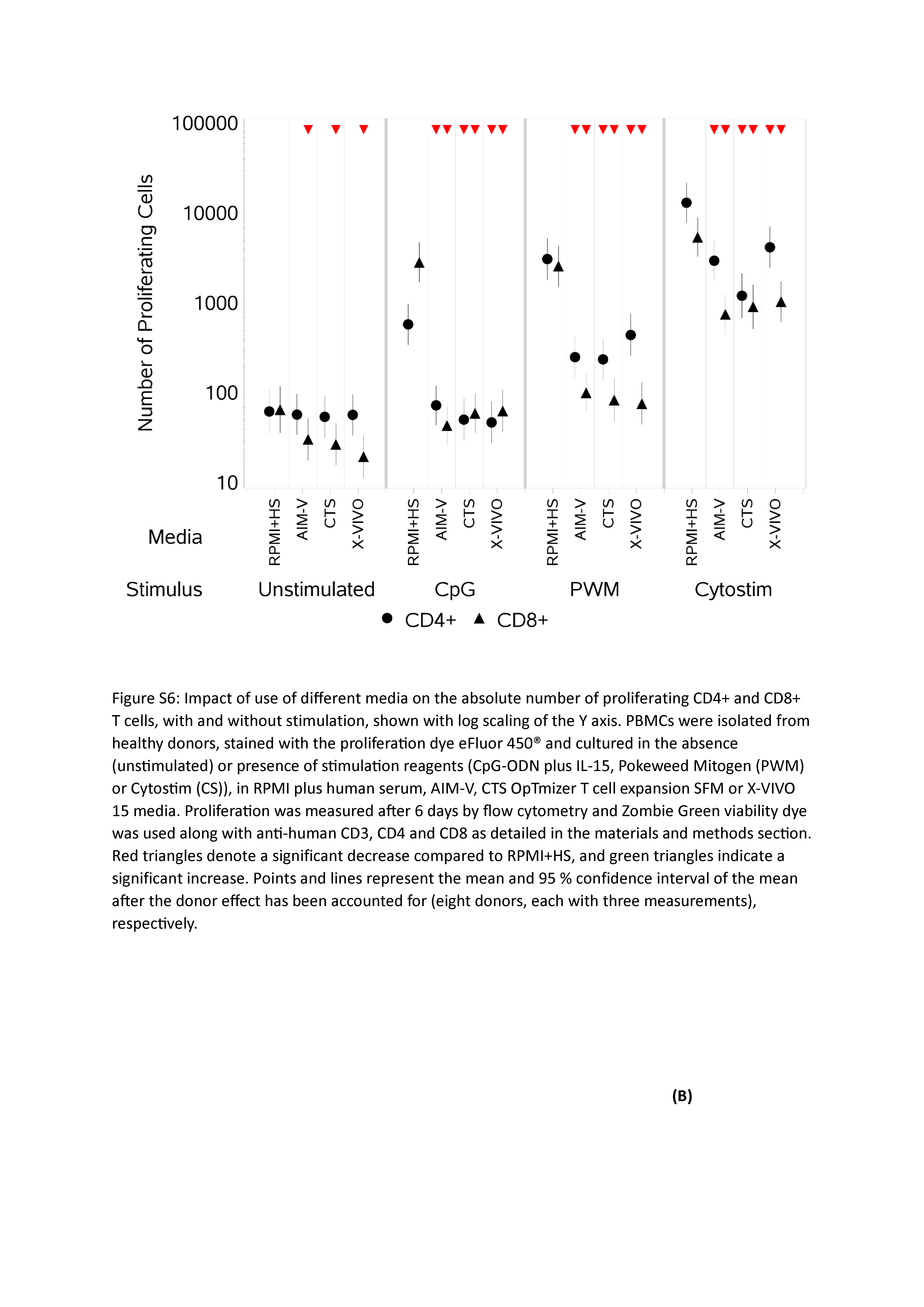

Supplement: Supplementary file 9 [file Image6.tiff]

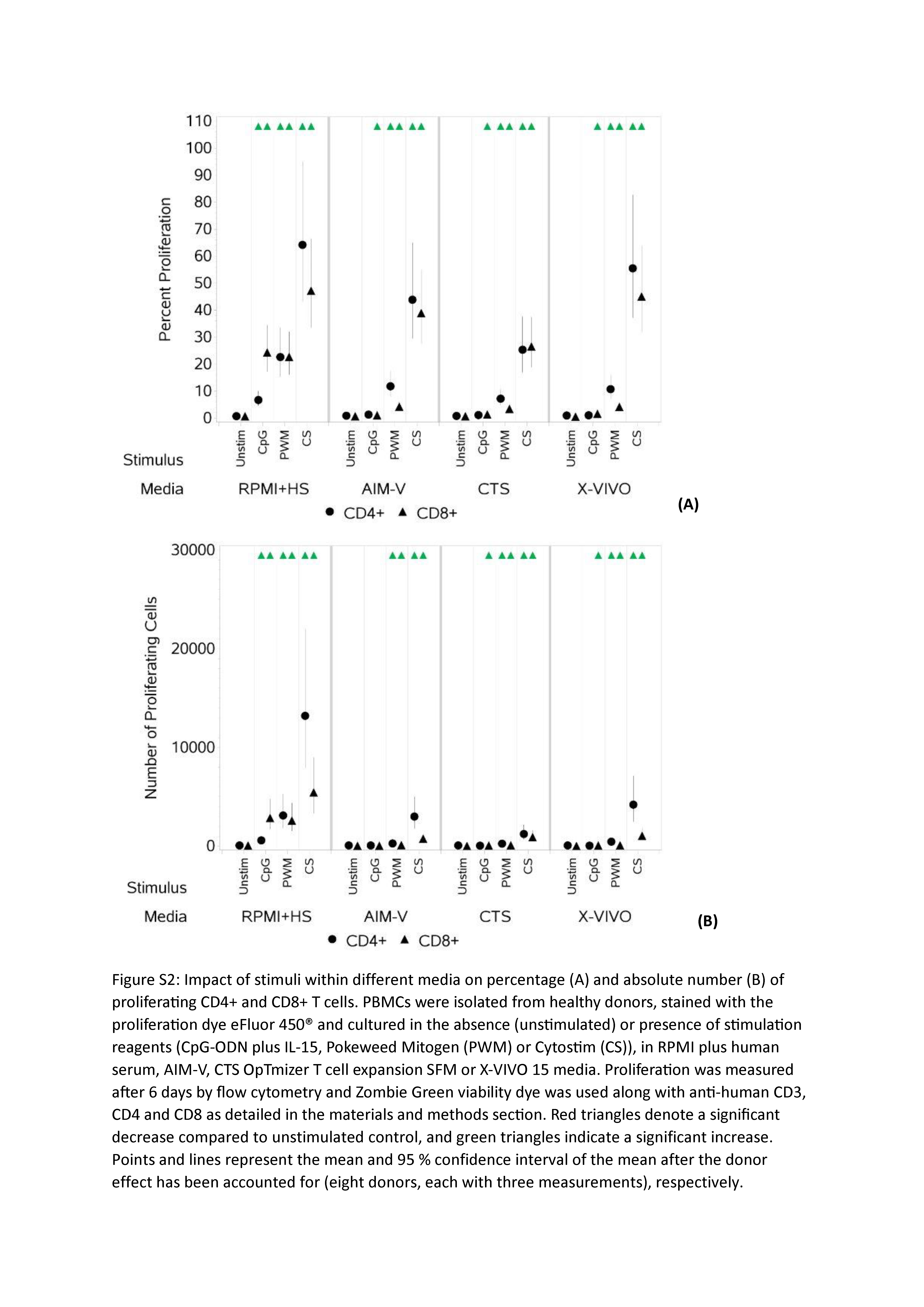

Supplement: Supplementary file 10 [file Image2.tiff]

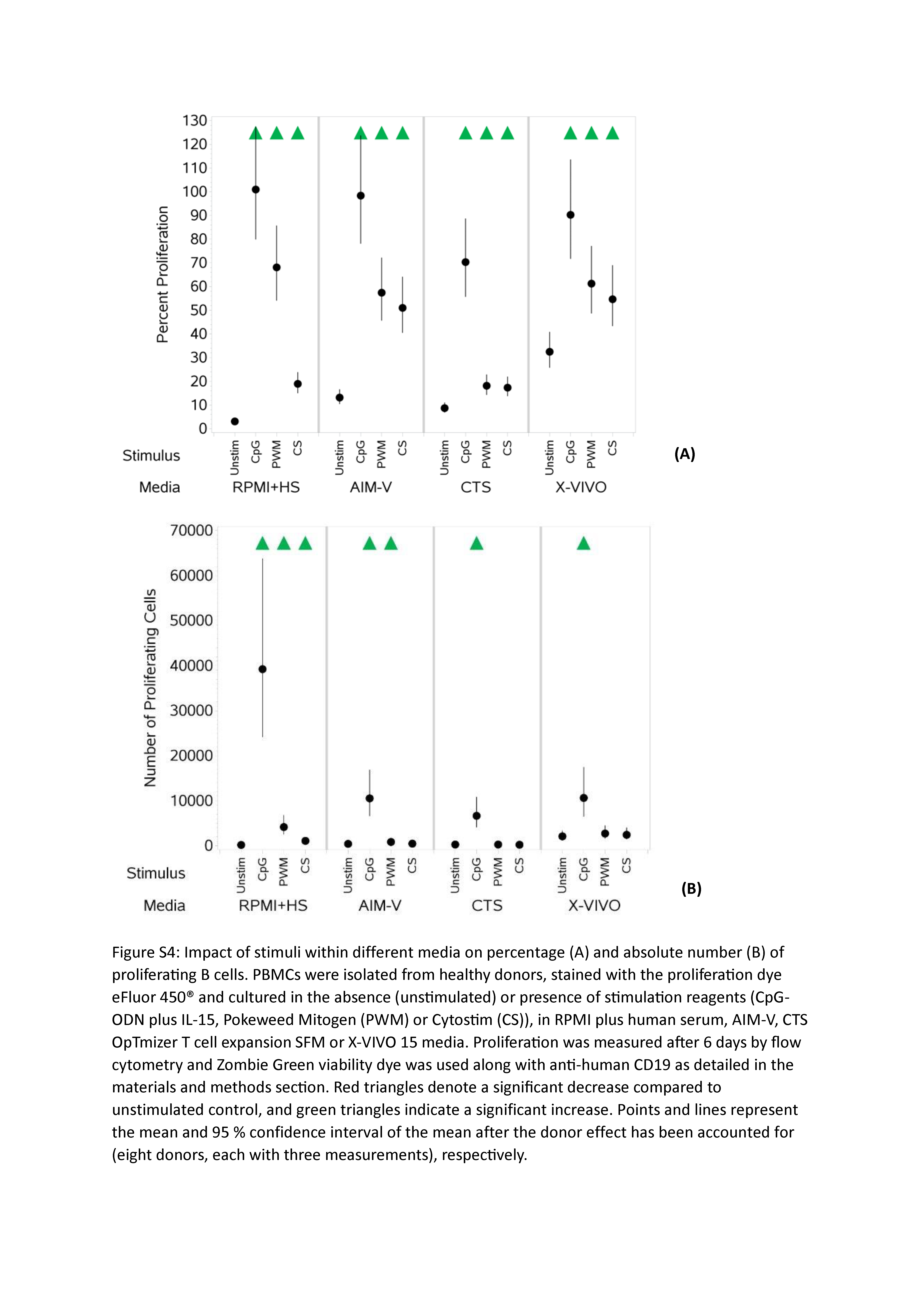

Supplement: Supplementary file 11 [file Image4.tiff]

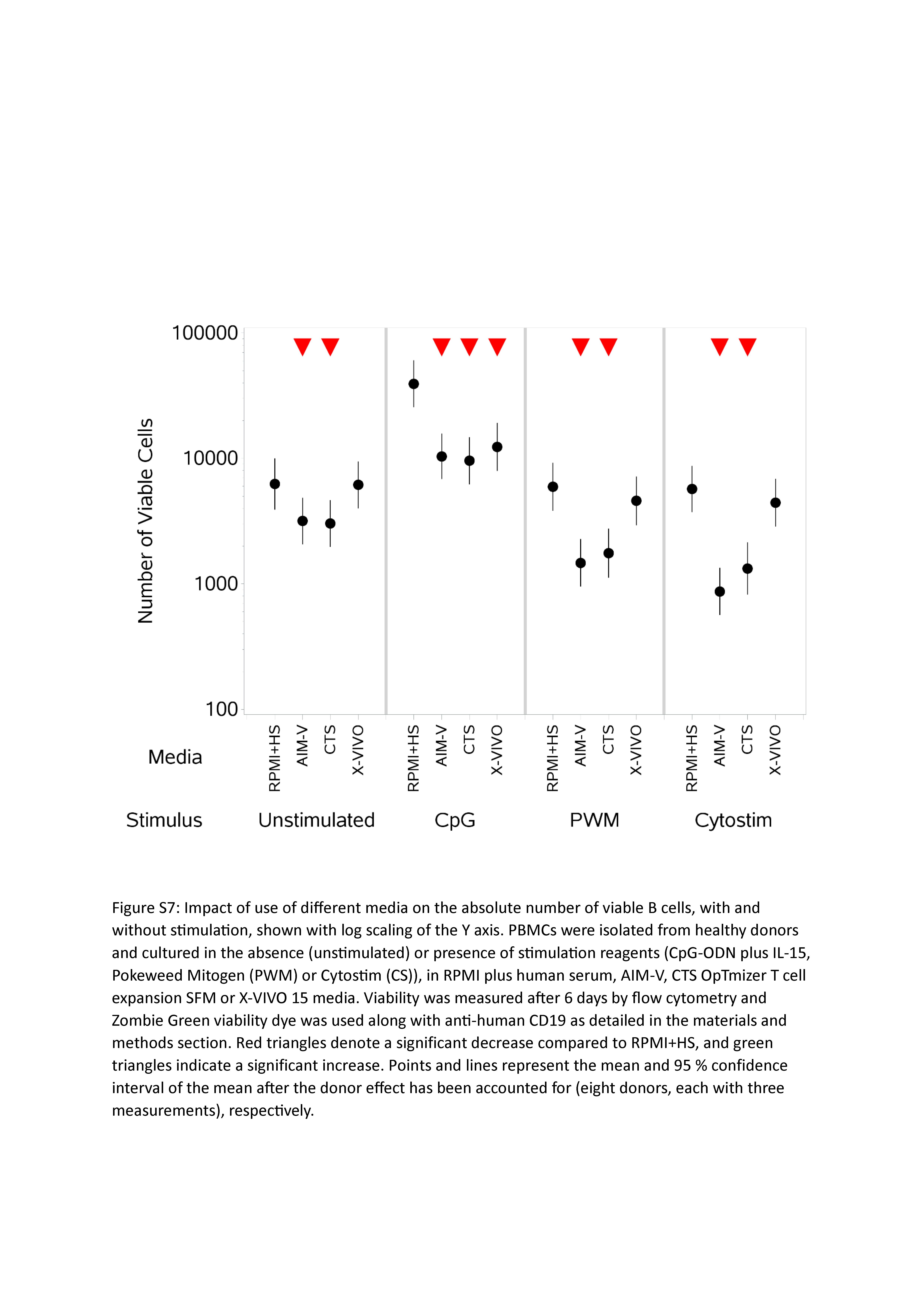

Supplement: Supplementary file 12 [file Image7.tiff]
